# The impact of follower leadership position on transformational leadership as moderator of the association between work-related ambiguity and job satisfaction

**DOI:** 10.3389/fpsyg.2022.970887

**Published:** 2022-09-23

**Authors:** Morten Birkeland Nielsen, Jørn Hetland, Anette Harris, Guy Notelaers, Johannes Gjerstad, Ståle Valvatne Einarsern

**Affiliations:** ^1^Department of Work Psychology and Physiology, National Institute of Occupational Health, Oslo, Norway; ^2^Department of Psychosocial Science, University of Bergen, Bergen, Norway; ^3^Department of Behavioural Sciences, Oslo Metropolitan University, Oslo, Norway

**Keywords:** managers, demands, resources, well-being, attitudes

## Abstract

This two-part study examined if the buffering effect of transformational leadership on the association between work-related ambiguity and job satisfaction is contingent upon whether a follower holds a formal leadership position him/herself. Data from two separate surveys were employed: Study 1: A sample of 845 respondents from Belgium. Study 2: A national probability sample of 1,608 Norwegian employees. Study 1 showed that task ambiguity had a significant negative relation with job satisfaction, but that transformational leadership did only buffer the association between task ambiguity and job satisfaction among employees holding a formal position as a supervisor or manager. Study 2 extended Study 1 by adjusting for age and job tenure of subordinates as a confounding variable. Study 2 confirmed that transformational leadership had a significantly stronger impact on the observed association between role ambiguity and job satisfaction among respondents holding a supervisor or manager position. In conclusion, when considering job satisfaction as an outcome of work-related ambiguity, transformational leadership is mainly beneficial for followers holding a formal supervisor or manager position themselves. Our findings thereby question assumptions about the general effectiveness of transformational leadership.

## Introduction

The main task of leaders is to set and achieve strategic goals for the organization while also motivating and supporting individuals within the group to successfully carry out assignments in service to those goals. Consequently, leadership is considered as a key factor regarding both the health, wellbeing, and work ability of employees and for the effectiveness and success of the organization. As for which types of leadership that are most beneficial for employees, several classifications and theoretical models of leadership practices have been described in the literature. For instance, in the well-known Full Range Model of Leadership (Avolio et al., 1999; Bass and Riggio, 2006), leadership is described on a spectrum ranging from passive, unproductive leadership styles, such as *laissez-faire* leadership and management by exception, through transactional forms of leadership, to active and constructive leadership styles, with transformational leadership assumed to be the most effective form. However, as the many contingency models of leadership show us, the efficacy of any given leadership style cannot be evaluated without knowledge of contextual factors, and to date, few studies have examined the impact of such factors on the effectiveness of transformational leadership (Oc, 2018).

Building on the relational identification theory (Sluss and Ashforth, 2008) and previous findings on the role of hierarchical role in the organization (Bruch and Walter, 2007), we suggest that follower leadership position, that is whether a subordinate occupies a formal supervisory or manager role in the organization themselves (Arvey et al., 2006), is one contextual factor that needs to be taken into account when evaluating the effectiveness of assumed effective leadership styles, such as transformational leadership. Specifically, this study aims (1) to determine whether transformational leadership moderates/buffers the well-documented association between work-related ambiguity with job satisfaction and (2) whether this buffering effect of transformational leadership is contingent upon whether a given employee holds a formal leadership, supervisor, or manager position him−/herself. The research aims were first addressed in a cross-sectional convenience sample from Belgium. To rule out possible cross-cultural biases and to strengthen the external validity of the findings, the research aim was thereafter replicated in a national probability sample of Norwegian employees controlling for the respondents’ job tenure.

### The moderating effect of transformational leadership

Job satisfaction is a measure of workers’ contentedness with their job, whether they like the job or individual aspects or facets of jobs, such as nature of work (Spector, 1997). Hence, job satisfaction is an important indicator of whether employees thrive at work. Role ambiguity and task ambiguity refer to the lack of clarity, certainty, and/or predictability one might have expected with regard to behavior in a job and is known to be strongly related to low job satisfaction (Abramis, 1994). Knowledge about factors that could reduce the negative effects of work-related ambiguity is therefore important. Syrek et al. (2013), pp. 254-255 present four reasons for why transformational leadership, defined as a form of leadership that involves binding people around a common purpose through reinforcing behaviors that follower gain from successfully achieving a task and from a reliance on intrinsic rewards (Oke et al., 2009), should be especially beneficial regarding maintaining, and even improving, job satisfaction among employees following stressful exposures such as ambiguity in one’s tasks or role. First, due to their ability to stimulate and inspire followers, transformational leaders may help redefine stressful situations, including the experience of ambiguity, and thereby reframe demands providing a new perspective on them (Lyons and Schneider, 2009; Niessen et al., 2017). That is, transformational leaders might temper the undesirable impact of ambiguity by inspiring employees to see any stress related exposures as challenges that can be achieved. Second, transformational leaders develop their followers through empowering the employees to work on their strengths and weaknesses (Bass, 1985). Hence, employees will more easily acquire new skills and behaviors necessary for coping with the experience ambiguity, potentially reducing employee stress and strengthening wellbeing (Syrek et al., 2013). Third, a transformational leader will respond to individual followers’ requirements and isthereby attentive to their needs for recognition, which should contribute to employees’ motivation and thereby wellbeing (Avolio and Bass, 1995). Fourth, the negative impact of a work-related ambiguity may be alleviated when followers understand and accept the reasons for the presence of the ambiguity (Bakker and Demerouti, 2007). As transformational leaders, through their visions and inspirational motivation, will communicate a clear sense of purpose, employees may comprehend and cope better with stressful situations, thus reducing the overall impact of the experienced ambiguity (Syrek et al., 2013). Based on the above reasoning, we suggest the following hypothesis:

*H1*: Transformational leadership moderates the relationship between role/task ambiguity and job satisfaction in that the magnitude of the association will be weaker among employees who experience high levels of transformational leadership compared to employees experiencing low levels of transformational leadership.

### The role of followers’ formal leadership position

As the middle manager position is more unpredictable and less routinized than that of other employees, the likelihood of exposure to hindrance demands such as role ambiguity and role conflict may be higher. Middle managers act as subordinates, equals, and superiors, and according to Embertson (2006), it is therefore common among many middle managers to experience and deal with role ambiguity since they have to balance multiple roles at the workplace. Thus, employees in a middle manager position may also be more in need of, and more positively affected by transformational leadership. Relational identification theory may contribute to explain why transformational leadership may have the strongest impact on middle managers.

According to relational identification theory, transformational leaders exert influence on followers through relational identification (Kark et al., 2003). However, the extent to which a follower will identify with the leader depends on the attractiveness or desirability of this role relationship (Sluss and Ashforth, 2008; Walumbwa and Hartnell, 2011). The more positive the evaluation of the role relationship, the more likely it is that the employee will include this relationship in his or her definition of self (Qu et al., 2015). This assumption is in line with self-concordance theory (Sheldon and Kasser, 1998; Sheldon and Elliot, 1999) which states that employees will be more satisfied and have a greater likelihood of goal accomplishment when their goals are driven by autonomous rather than controlled (external reinforcement) motives (Downes et al., 2017). Autonomous motives “include pursuing goals because they are fun (intrinsic motives) or because of the fundamental belief that the goal is an important one to accomplish (identified motives)” (Downes et al., 2017, p. 198). Hence, relational identification theory suggests that the closer a need or motivator is perceived to be in relation to oneself, the more powerful it is as a motivating factor (Sluss and Ashforth, 2008). That is, when people perceive a message source to be comparable to themselves, then this source will have a stronger persuasive influence (Kelman, 1961). Regarding the influence of leadership, the stronger a follower relates to, or identifies with, the leader, the more likely the follower is to be motivated and influenced by the leader. According to Sluss and Ashforth (2008), subordinate’s identification with the follower-manager role relationship (i.e., relational identification) should converge with the follower’s organizational identification. Consequently, it seems reasonable that followers who are directly involved in leadership processes themselves, such as line, project, and middle supervisors or managers, should relate more strongly to, and thereby be more influenced by, their immediate leader. Taken together, the above arguments suggest that a transformational leader should exert a stronger influence on followers holding a formal position as a supervisor or manager than on employees in general by making the perceived desirability and attractiveness of the role relationship greater for the former, and thereby leading him or her to appreciate or admire the leader, internalizing his or her goals, values, and beliefs. As employees not holding a position as a supervisor or manager are less involved in organizational decisions, they should also be less likely to relationally identify with the leader and thereby define themselves in terms of the leader–follower role relationship.

Middle management works across organizational networks, motivating communication and creating an environment that encourages the sharing information (Potthoff, 2004). The formal position in the organizational hierarchy may therefore itself also influence the efficacy of transformational leaders. As suggested by Bruch and Walter (2007), pp. 721–722, “explicitly incorporating hierarchical factors in theoretical and empirical models of transformational leadership emergence and effectiveness may prove insightful and lead to a more precise depiction of the transformational leadership process.” First, transformational leadership have been found to be more prevalent at higher organizational levels compared to the lower levels (Edwards and Gill, 2012), whereas lower-level supervisors/managers are more likely to display transactional leadership style than upper-level leaders (Lowe et al., 1996). As for specific transformational behaviors, Bruch and Walter (2007) found in a study of 448 leaders that idealized influence and inspirational motivation were more widespread among upper rather than middle-level leaders. Secondly, being the link between upper management and employees at lower levels, middle supervisors are also more likely to be influenced and inspired by transformational leadership, since upper managers seem to be in a better position to display visionary and charismatic leadership conduct (Edwards and Gill, 2012).

The above reasoning suggests that transformational leadership is likely to exert a stronger influence on subordinates holding a formal supervisor or manager position, and that the effectiveness of transformational leadership thereby is dependent upon subordinate supervisor/manager role as a contextual factor. We suggest that subordinates in such a role themselves will be especially important regarding the effectiveness of transformational leadership and therefore how transformational leadership may buffer the impact of role and task ambiguity. To provide empirical evidence for the subordinate being in a supervisor or manager role as a contextual factor in evaluation of transformational leadership effectiveness, the current study will determine whether the buffering effect of transformational leadership on the association between task and role ambiguity and job satisfaction is conditioned by subordinate position in the organizational hierarchy. The following hypothesis will be tested:

*H2*: The buffering effect of transformational leadership on the association between role/task ambiguity and job satisfaction is strongest among followers holding a formal position as a supervisor or manager themselves (compared to followers without such a formal position).

Understanding of cross-cultural differences in transformational leadership behavior is incomplete (Crede et al., 2019) and there is a shortage of studies comparing the effectiveness of transformational leadership across different cultures. To add to the knowledge on the role of culture in transformational leadership, the hypothesis will be investigated in two European countries with relatively different cultural characteristics, namely Belgium and Norway. According to Hofstede (2001), Belgium is characterized by very high scores on individualism, uncertainty avoidance, and long-term orientation, and by high scores on power distance, masculinity, and indulgence. In contrast, Norway is characterized by low scores on power distance, masculinity, and long-term orientation, and by medium-to-high scores on individualism, uncertainty avoidance, and indulgence. Hence, it is not unlikely that leadership may be executed, and perceived, differently in these two countries.

## Materials and methods

### Study 1: Belgian sample

#### Sample and procedure

Study 1 is based on a convenience sample of 845 respondents recruited from six Belgian organizations between 2015 and 2017. The data collection was conducted by a consulting firm on behalf of Human Resource Management services in Belgium. Most respondents answered the questionnaire through an electronic form, although pen-and-pencil questionnaires were available for respondents who preferred this approach. Participation in the survey was informed, anonymous, confidential, and voluntary. The response rate for the survey was not available.

The sample comprised somewhat more men (54%) than women (46%); 3% of the respondents were less than 25 years old, 28% were between 25 and 34 years old, 27% were between 35 and 44 years old, 31% were between the age of 45 and 54, and 11% were between 55 and 65 years old. According to the coding of economics activities (NACE-BEL), 81% were employed in manufacturing industries, 4% in the production and distribution of gas and electricity, while 15% was involved in scientific or technical activities. Altogether 22% of the respondents had a formal leadership role.

#### Inventories

*Job satisfaction* was measured with five items from The Short Inventory to Monitor Psychosocial Hazards (SIMPH 5; Notelaers et al., 2007), an indicator of global job satisfaction where respondents are asked to indicate agreement with the items by answering “no” or “yes.” Example items are “mostly, I am pleased to start my day’s work” and “I really enjoy my work.” The responses were summarized into an overall scale that had acceptable internal consistency (Cronbach’s alpha: 0.82).

As an indicator of job demands, *task ambiguity* was measured with three items from the SIMPH (Notelaers et al., 2007). The response scale was 4-point with categories ranging from “never” to “always.” A high score indicates that the respondent finds it difficult to adapt to changes in work tasks. Example items are “do you find it difficult to adapt to changes in your tasks?” and “do the changes in your tasks cause you problems?” Cronbach’s alpha was 0.80.

*Global Transformational Leadership Scale* (GTL; Carless et al., 2000) was included as an indicator of *transformational leadership*. By including seven items, this short scale measures transformational leadership as a unified construct, and is intended to be a global assessment of perceived transformational leadership of the immediate leader (Carless et al., 2000). The items capture seven leadership behaviors: (i) Communicates a clear and positive vision, (ii) develops staff, (iii) supports staff, (iv) empowers staff, (v) is innovative, (vi) leads by example, and (vii) is charismatic (e.g., “my leader fosters trust, involvement and co-operation”). All items were answered on a 7-point scale ranging from “strongly disagree” to “strongly agree.” The GTL has high convergent validity with other indicators of transformational leadership such as the Multifactor Leadership Questionnaire (MLQ) and the Leadership Practices Inventory (LPI) (Carless et al., 2000). Cronbach’s alpha for the GTL was 0.93 in Study 1.

Formal position as a supervisor was assessed with a single item: “Are you a supervisor?” Response categories were: “no” and “yes, I have responsibility over other collaborators’.”

### Study 2: Norwegian sample

#### Sample and procedure

A random and nationally probability sample of 5,000 employees was drawn from The Norwegian Central Employee Register by Statistics Norway. This is the official register of all Norwegian employees, as reported by employers. The inclusion criteria were adult employees within the age range 18–60, being employed in a Norwegian enterprise. Questionnaires were distributed in spring 2015. The overall response rate was 32%. This study includes 1,608 questionnaires that were satisfactorily completed. The survey had approval from the “Regional Committee for Medical Research Ethics for Eastern Norway” (approval 2014/1725). All respondents provided informed consent to participate in the survey and the responses were treated anonymously.

The study sample consisted of more women (52%) than men (48%). The mean age was 45.19 (*SD =* 10.04) years with a range from 21 to 61. In total, 53% were married, 26% were common-law partners, 14% were unmarried, and 7% were widowed, separated, or divorced. Altogether 9% had primary school as the highest educational level, 31% had high school, 32% had lower-level university, while 28% were graduates or postgraduates. The average job tenure was 11.3 years. Thirty-six percent of the participants had a leadership role that included personnel responsibilities.

#### Inventories

Job satisfaction was measured with the short version of The Job Satisfaction Scale (Brayfield and Rothe, 1951) as presented by Hetland et al. (2008) assessed job satisfaction among the respondents. Example items are: “I feel fairly satisfied with my present job” and “most days I am enthusiastic about my work.” Responses were given on a 5-point Likert scale where 1 = “strongly disagree’” and 5 = “strongly agree.” Cronbach alpha in Study 2 was 0.81.

A previously validated three items instrument from the QPS_Nordic_ (Dallner et al., 2000; Wannstrom et al., 2009) measured *role ambiguity*. Example items are “do you know what your responsibilities are?” and “do you know exactly what is expected of you at work?” Respondents responded on a 5-point Likert scale ranging from “very seldom or never” to “very often or always.” The response scale was reversed in the current study as they were originally ordered to designate role *clarity*. The inventory had a Cronbach’s alpha of 0.80 in this study.

As in Study 1, transformational leadership was assessed with *The Global Transformational Leadership Scale* (Carless et al., 2000) 1. All items were answered on a 5-point Likert scale ranging from”never” to”very often or always.” A previous study has found that this Norwegian translation has acceptable psychometric properties (Nielsen et al., 2019). Cronbach’s alpha in Study 2 was 0.94.

Formal position as a supervisor was assessed by a single item question asking, “Do you have position as a supervisor?” Response categories were “no” and “yes.”

### Statistical analysis

Statistical analyses were conducted using IBM SPSS Statistics 25.0 and the Process 4.0 macro script for SPSS (Hayes, 2013). A two-way interaction between the variables was tested in the linear regression analysis by adding an interaction term between job demands (task/role ambiguity) and transformational leadership (e.g., job demands × transformational leadership). Three-way interactions were tested by adding an interaction term between job demands, transformational leadership, and leadership position (job demands × transformational leadership x leadership position). Continuous were centered in the analyses. The level of significance was *p* < 0.05.

### Control variables

Several individual follower characteristics have been associated with perceptions of leadership and the outcomes of leadership. In a combined meta-analysis and primary investigation of the role of followers in ratings of leadership behavior, gender appeared to have a substantial impact on ratings of transformational leadership (Wang et al., 2019). In addition, age, gender, and job tenure have been found to influence ratings of job satisfaction (Bedeian et al., 1992). To rule out plausible confounding effects of follower characteristics on the main study association, the analyses will be adjusted for age and gender in Studies 1 and 2. Study 2 will extend Study 1 by also adjusting for job tenure.

## Results

### Study 1

Descriptive statistics and intercorrelations for all variables in Study 1 are presented in Table 1. The directions and magnitude of all correlations were as to be expected. Task ambiguity was negatively, whereas transformational leadership was positively, associated with levels of job satisfaction. Task ambiguity was negatively related to transformational leadership. Having a formal position as a supervisor or manager was positively correlated with male gender, job satisfaction, and transformational leadership, but not related to task ambiguity.

**Table 1:** Means, SD, and correlations for variables in Study 1.

	Variable	Range	M	SD	1	2	3	4	5
1	Position as a supervisor	0–1	0.22	0.41	–				
2	Gender (ref. woman)	0–1	0.53	0.50	0.10^**^	–			
3	Job satisfaction	0–1	0.74	0.33	0.20^***^	−0.05	–		
4	Task ambiguity	1–4	1.63	0.51	0.04	0.06	−0.22^***^	–	
5	Transformational leadership	1–7	4.32	1.47	0.14^***^	0.03	0.40^***^	−0.15^***^	–

Reference category gender: “Woman.” Reference category position as a supervisor: “No.”

** *p* < 0.01;

*** *p* < 0.001.

**Figure 1:**
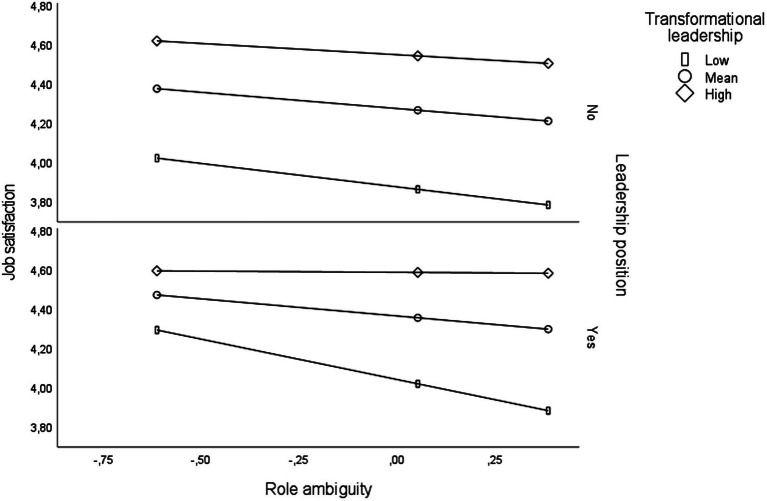
Three-way interaction between task ambiguity, transformational leadership, and position as a supervisor regarding job satisfaction adjusted for age and gender (Study 1).

Findings from the analyses of main, two-, and three-way interaction effects in Study 1 are presented in Table 2. The results from the full model show that task ambiguity (*B* = −0.12; *p* < 0.001), transformational leadership (*B* = 0.08; *p* < 0.001), and having a leadership position (*B* = 0.15: *p* < 0.001) were directly associated with job satisfaction. There were no significant two-way interactions, but a significant three-way interaction (*B* = 0.08; *p* < 0.05) showed that the buffering effect of transformational leadership on the association between task ambiguity and job satisfaction was contingent upon whether the respondent had a formal position as a supervisor or manager. As illustrated in Figure 1, the findings show that the interaction between task ambiguity and transformational leadership was only significant among respondents holding a supervisor or manager position. Specifically, the findings indicate that transformational leadership attenuates the magnitude of association between task ambiguity and job satisfaction among respondents holding a supervisor or manager position (*B* = 0.07; *p* < 0.05) but does not impact this association among respondents without such a position (*B* = 0.01; *p* > 0.05). The interaction term for the three-way interaction indicates that the difference in effect between the two groups was significant. The predictor variables explained 21.8% of the variance in job satisfaction. The overall model was significant (*F* = 28.87; *DF* = 8/827; *p* < 0.001).

**Table 2:** Two-and three-way interactions between role ambiguity, transformational leadership, and position as a supervisor regarding job satisfaction in Study 1 (*N* = 845); *R*^2^ = 0.22.

Variables	*B*	SE B	*t*
Gender	−0.03	0.02	1.59
Task ambiguity	−0.12	0.02	−5.65^***^
Transformational leadership	0.08	0.01	10.98^***^
Task ambiguity × transformational leadership	0.01	0.01	0.91
Position as a supervisor	0.15	0.03	5.71^***^
Task ambiguity × position as a supervisor	−0.05	0.06	−0.96
Transformational leadership × position as a supervisor	−0.03	0.02	−1.80
Task ambiguity × transformational leadership × position as a supervisor	0.08	0.03	2.38^*^
Constant	0.80	0.03	24.30^***^

*B* is unstandardized beta coefficients.

* *p* < 0.05;

*** *p* < 0.001.

### Study 2

Means, standard deviations, and bivariate correlations for all study variables are presented in Table 3. The directions and magnitude of all correlations were as to be expected. Role ambiguity was negatively, whereas transformational leadership was positively, related to job satisfaction. Transformational leadership was negatively correlated with role ambiguity. Having a position as a supervisor/manager was positively correlated with being male, higher job satisfaction but was not associated with neither transformational leadership nor role ambiguity. Job tenure was positively associated with job satisfaction, negatively related to role ambiguity, and unrelated to transformational leadership.

**Table 3:** Means, SD, and correlations for variables in Study 2.

	Variable	Range	*M*	*SD*	1	2	3	4	5	6	7
1	Position as a supervisor	0–1	0.36	0.48	–						
2	Gender	0–1	0.52	0.50	−0.20^***^	–					
3	Age	21–59	45.52	16.98	0.08^**^	−0.03	–				
4	Job tenure	0–41	11.32	9.51	0.08^***^	0.09^***^	0.32^***^	–			
5	Job satisfaction	1–5	4.22	0.71	0.08^**^	0.04	0.06^*^	−0.34^***^	–		
6	Role ambiguity	1–4	1.61	0.56	−0.04	0.02	−0.16^***^	0.22^***^	−0.29^***^	–	
7	Transformational leadership	1–5	3.67	0.84	0.05	0.04	−0.01	−0.22^***^	0.47^***^	−0.28^***^	–

Reference category gender: “Man.” Reference category position as a supervisor: “No.”

* *p* < 0.05;

** *p* < 0.01;

*** *p* < 0.001.

**Figure 2:**
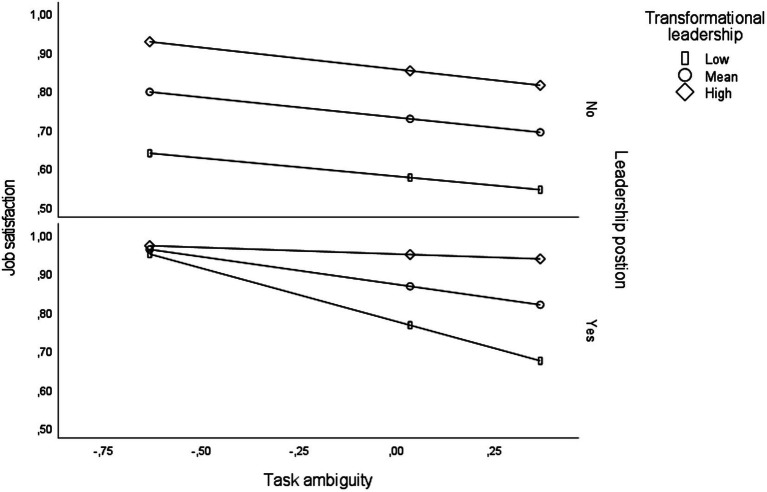
Three-way interaction between role ambiguity, transformational leadership, and position as a supervisor regarding job satisfaction adjusted for age, gender, and job tenure (Study 2).

Findings from the analyses of two-and three-way interaction effects are displayed in Table 4. The analyses were adjusted for age, gender, and job tenure. Regarding main effects, the estimates showed that role ambiguity (*B* = −0.19; *p* < 0.001) and transformational leadership (*B* = 0.36; *p* < 0.001) were significantly associated with levels of job satisfaction. Further, a positive beta-coefficient showed that respondents with a formal position as a supervisor/manager had higher levels of job satisfaction compared to non-supervisors/managers (*B* = 0.10; *p* < 0.01). A two-way interaction effect was established between role ambiguity and transformational leadership (*B* = 0.13; *p* < 001). This interaction analysis showed that the negative association between role ambiguity and job satisfaction was most profound among respondents reporting high levels of transformational leadership. Specifically, as exhibited in Figure 2, the analyses of the three-way conditional effect (*B* = 0.16; *p* < 0.05) showed that the interaction between role ambiguity and transformational leadership regarding job satisfaction was significant among respondents holding a position as a supervisor/manager (*B* = 0.23; *p* < 0.001), but only borderline significant among respondents not holding a supervisor/manager position (*B* = 0.07, *p* ≤ 0.05). Altogether, the predictor variables explained 26.3% of the variance in job satisfaction. The overall model was significant (*F* = 54.65; *DF* = 10/1530; *p* < 0.001).

**Table 4:** Two-and three-way interactions between role ambiguity, transformational leadership, and position as a supervisor regarding job satisfaction in Study 2 (*N* = 1,541); *R*^2^ = 0.26.

Variables	*B*	SE B	*T*
Age	0.00	0.00	0.87
Gender	0.05	0.03	1.44
Job tenure	−0.00	0.00	1.26
Role ambiguity	−0.19	0.03	−6.29^***^
Transformational leadership	0.36	0.02	18.27^***^
Role ambiguity × transformational leadership	0.13	0.03	3.98^***^
Position as a supervisor	0.10	0.04	2.97^**^
Role ambiguity × position as a supervisor	−0.04	0.06	−0.60
Transformational leadership × position as a supervisor	−0.07	0.04	−1.74
Role ambiguity × transformational leadership × position as a supervisor	0.16^*^	0.07	2.18^*^
Constant	4.07^***^	0.09	44.85

*B* is unstandardized beta coefficients.

* *p* < 0.05;

** *p* < 0.01;

*** *p* < 0.001.

## Discussion

Although evidence suggests that transformational leadership is beneficial for follower’s wellbeing (Arnold, 2017), the overall effectiveness of this kind of leadership practice has also been questioned and criticized (van Knippenberg and Sitkin, 2013; Mhatre and Riggio, 2014). The findings of this study provide empirical support for the critique related to transformational leadership by examining follower position as a supervisor or manager as a contextual factor that determines for whom transformational leadership may be most beneficial. Based on data from large and heterogeneous samples from two different countries, our findings showed that transformational leadership only had a buffering effect regarding the impact of work-related ambiguity on job satisfaction for employees holding a position as a supervisor or manager themselves. Transformational leadership did not moderate the association between ambiguity and job satisfaction among employees without a formal position as a supervisor or manager. The findings were not influenced by potential confounding factors such as age, gender, and job tenure among followers and were consistent across the two samples and two concepts and measures of ambiguity. Hence, the findings indicate that having a formal leadership role at the workplace is a contextual factor that determines when and for whom transformational leadership is effective.

There have been a number of previous studies that have investigated transformational leadership across organizational levels (see Edwards and Gill, 2012 for an overview). However, these studies have mainly examined differences in the exertion of transformational leadership behavior between leaders at different hierarchical levels rather than assessing the effectiveness regarding reducing the impact of work-related stressors on outcomes. An exception is a study of 367 managers from 38 United Kingdom organizations in the manufacturing industries which found that transformational leadership was equally effective across hierarchical levels in organizations (Edwards and Gill, 2012). However, this study was limited to perceived general effectiveness and did not provide any information about specific work stressors or employee outcomes. Hence, the current study extends previous research by determining when and for whom transformational leadership is most beneficial.

As discussed in the introduction, the differential effect of position as a supervisor or manager on the impact of transformational leadership on the outcomes of work-related ambiguity established in the current study may be explained by the relational identification theory. Subordinates holding such a position should be more likely to identify with their immediate leader and therefore be more responsive to transformational leadership through a relational identification process. Higher-level managers have been suggested to exhibit transformational leadership to a greater extent than do middle managers (Lowe et al., 1996). For instance, upper managers have been proposed to have better opportunities than middle managers to provide intellectual stimulation (Bruch and Walter, 2007). Due to their increased autonomy, higher-level leaders may have liberty to probe innovative solutions and to include their followers in the pursuit of novel approaches, whereas it may be more challenging for middle managers to approach work problems in innovative ways, as the tasks of their followers are more clearly pre-defined and because they lack the power to endorse innovative processes (Bruch and Walter, 2007). Supporting this assumption, it has been shown that transformational leadership is more frequently displayed at higher levels in organizations while lacking at middle and lower-level (Edwards and Gill, 2012). This suggests that the relational identification may be especially strong in the relationship between an upper and middle manager, whereas it is less pronounced in the relationship between middle managers and their subordinates. Consequently, when experiencing work-related ambiguity, middle managers have more positive resources to draw upon as higher-levels leaders are more likely to exhibit a transformational leadership style which, as shown in this study, is beneficial with regard to maintaining job satisfaction also in cases of high ambiguity.

The finding that holding a position as a supervisor/manager is a contextual factor that determines the effectiveness of transformational leadership was consistent across the two samples included in this study, even if the cultures of Belgium and Norway are not equal regarding cultural characteristics (Hofstede, 2001). Hence, due to the cultural differences between the study samples, our findings are probably not a consequence of cultural characteristics and are indeed likely to be present in most countries and organizations, at least in western cultures.

### Methodological considerations

This study has some important strengths and limitations that should be considered in the interpretation of the findings. As for strengths, our study includes two large and heterogeneous samples from Norway and Belgium. The Norwegian sample is from a national probability survey of the general working population. Thus, it should be possible to generalize our findings to most occupations and industries. The internal validity should be strengthened by the fact that the study variables were assessed with psychometrically sound and valid instruments.

As for limitations, the response rate was not available for the Belgian sample, and the response rate of 32% in the Norwegian sample is low, yet in line with the average rate in survey research (Stedman et al., 2019). Still, with 68% non-responders, we cannot rule out that the study sample differs from non-responders on demographic characteristics or on the main study variables. All data were collected using self-report methods. This amplifies the risk for common method variance and response set tendencies. There is a risk of single source bias in the form of overlapping variability as all data were based on employee reports. Furthermore, the cross-sectional design of the study does not permit any conclusion about the causal order of variables. Hence, even though the study is founded on the theoretical assumption that transformational leadership moderates the relation between work-related ambiguity and job satisfaction, other causal associations may exist. To determine causality, our study should be replicated using longitudinal or experimental research designs. However, it should be noted that longitudinal designs have their own limitations (e.g., inadequate time-lags, unmeasured third variables, and so on), and may therefore be only marginally better than cross-sectional designs (Spector, 2019): “The knowledge that many pairs of variables are associated, even without knowing the causal connections, is extremely valuable as a basis for theory and the target of intervention” (Spector, 2019, p. 136).

Transformational leadership was assessed with the Global Transformational Leadership Scale (Carless et al., 2000). Although this scale is a frequently used indicator of transformational leadership, it does not overlap perfectly with the dimensions described in the full range of leadership model by Bass and Avolio (2004). This study was therefore limited to examining transformational leadership as a second-order global construct rather than investigating the dimensionality of this type of leadership. Comparisons show that the Global Transformational Leadership Scale correlates strongly with other measures of transformational leadership such as the MLQ (Bass and Avolio, 2004) and the Leadership Practices Inventory (Kouzes and Posner, 1990), thus still indicating high convergent validity (Carless et al., 2000).

### Implications, and directions for future research

As we have shown that transformational leadership mainly is beneficial for alleviating the outcome of stressors for followers holding a superior position themselves, our study support concerns that have been raised about the overall success of transformational leadership (van Knippenberg and Sitkin, 2013; Mhatre and Riggio, 2014). An important implication for research is therefore that the followers’ hierarchical position in the organization should be taken into consideration when interpreting research findings on transformational leadership.

The findings have matching implications for the development and training of leaders. Given the favorable impact of transformational leadership (Judge and Piccolo, 2004), organizations should aim at strengthening the ability of, and opportunities for, transformational leadership among upper and middle management. One way of facilitating transformational leadership practices among middle managers could be to methodically reduce hierarchical constraints at lower managerial levels. By this kind of empowering, middle managers may become armed with better prospects for charismatic and visionary action, and their subordinates’ context may become more favorable for the effects of such leadership (Bruch and Walter, 2007). On the other hand, as noted, it may be that more transactional forms of leadership simply are more effective among lower-level employees and that organizations should allow for transactional leadership behavior to become more effective. That is, while higher-level leaders may inspire and support their subordinate managers to cope with, and grow from, role ambiguity, lower-level managers should focus on reducing role ambiguity in the first place. Alternatively, in line with the perspective on flexible leadership (Yukl and Mahsud, 2010), it may be that leaders need to adapt their style or approach in response to different groups of employees. That is, there may not be one single approach to leadership that is effective in all situations and for all employees.

This study was limited to examining the buffering effects of transformational leadership on the associations between work-related ambiguity and job satisfaction. Upcoming research should extend our study by the inclusion of other job stressors, and other outcome variables, to determine whether, and how, they also may be influenced by transformational leadership and subordinate position as a supervisor or manager. The role of subordinate position in this respect should also be examined with regard to other indicators of leadership, including constructive forms such as instrumental (Antonakis and House, 2014), authentic (Avolio and Gardner, 2005), and ethical leadership (Brown et al., 2005), as well as destructive forms such as *laissez-faire* leadership (Skogstad et al., 2007), and abusive supervision (Tepper et al., 2017).

## Conclusion

The finding that the effectiveness of transformational leadership is dependent upon the followers’ own formal position as a supervisor or a manager is a significant and noteworthy finding that extends previous knowledge on this kind of leadership practice. As our results suggest that transformational leaders exert their effects in complex processes where the effect depends on hierarchical positions of the follower, the study adds to the emerging debate as to whether transformational leadership is uniformly beneficial across all contexts and outcomes (Nielsen and Daniels, 2016). Furthermore, having shown that being a leader oneself, i.e., the position in the organization, has a substantive role regarding the effects of transformational leadership, this means that the variable should not simply be adjusted for in research as this can lead to removing the effects of the key constructs one wishes to study.

## Data availability statement

The raw data supporting the conclusions of this article will be made available by the authors, without undue reservation.

## Ethics statement

The studies involving human participants were reviewed and approved by Regional Committee for Medical Research Ethics for Eastern Norway. The patients/participants provided their written informed consent to participate in this study.

## Author contributions

MN in initiated the study, conducted analyses, and was responsible for writing the manuscript. JH, AH, JG, GN, and SE participated in the idea development, contributed to the structure and content, and read all versions of the manuscript. GN was responsible for the data collection in Study 1. JG and MN were responsible for the data collection in Study 2. All authors contributed to the article and approved the submitted version.

## Funding

This work was supported by the Norwegian Research Council (grant nos. 250127/237777).

## Conflict of interest

The authors declare that the research was conducted in the absence of any commercial or financial relationships that could be construed as a potential conflict of interest.

## Publisher’s note

All claims expressed in this article are solely those of the authors and do not necessarily represent those of their affiliated organizations, or those of the publisher, the editors and the reviewers. Any product that may be evaluated in this article, or claim that may be made by its manufacturer, is not guaranteed or endorsed by the publisher.
